# Fast Quantum Approach for Evaluating the Energy of Non-Covalent Interactions in Molecular Crystals: The Case Study of Intermolecular H-Bonds in Crystalline Peroxosolvates

**DOI:** 10.3390/molecules27134082

**Published:** 2022-06-24

**Authors:** Alexander G. Medvedev, Andrei V. Churakov, Mger A. Navasardyan, Petr V. Prikhodchenko, Ovadia Lev, Mikhail V. Vener

**Affiliations:** 1 Kurnakov Institute of General and Inorganic Chemistry, Russian Academy of Sciences, Leninskiy Prospekt 31, 119991 Moscow, Russia; mag@igic.ras.ru (A.G.M.); churakov@igic.ras.ru (A.V.C.); navasardyan@igic.ras.ru (M.A.N.); prikhman@gmail.com (P.V.P.); 2 The Casali Center of Applied Chemistry, The Institute of Chemistry, The Hebrew University of Jerusalem, Jerusalem 9190401, Israel; ovadia@mail.huji.ac.il

**Keywords:** peroxosolvates, macrocyclic ether, amino acid, periodic DFT computations, bifurcated H-bonds, multicomponent crystals, B3LYP vs. PBE-D3

## Abstract

Energy/enthalpy of intermolecular hydrogen bonds (H-bonds) in crystals have been calculated in many papers. Most of the theoretical works used non-periodic models. Their applicability for describing intermolecular H-bonds in solids is not obvious since the crystal environment can strongly change H-bond geometry and energy in comparison with non-periodic models. Periodic DFT computations provide a reasonable description of a number of relevant properties of molecular crystals. However, these methods are quite cumbersome and time-consuming compared to non-periodic calculations. Here, we present a fast quantum approach for estimating the energy/enthalpy of intermolecular H-bonds in crystals. It has been tested on a family of crystalline peroxosolvates in which the H∙∙∙O bond set fills evenly (i.e., without significant gaps) the range of H∙∙∙O distances from ~1.5 to ~2.1 Å typical for strong, moderate, and weak H-bonds. Four of these two-component crystals (peroxosolvates of macrocyclic ethers and creatine) were obtained and structurally characterized for the first time. A critical comparison of the approaches for estimating the energy of intermolecular H-bonds in organic crystals is carried out, and various sources of errors are clarified.

## 1. Introduction

A quantitative description of intermolecular (non-covalent) interactions in the gas phase can be achieved using a variety of theoretical approaches: NBO analysis [1], SAPT [2], etc. [3,4,5]. There is a large amount of experimental data from which “reference” values of the energy/enthalpy of intermolecular interactions can be obtained [6,7,8,9,10]. Molecular structure, chemical bond theory, and “standard” quantum chemical approaches focus on non-periodic systems. Their “generalization” to molecular crystals is not straightforward because of the need to explicitly take into account the periodic boundary conditions [11,12]. The identification and quantitative description of intermolecular interactions in crystals is a non-trivial task, both experimentally and theoretically. An exception are molecular crystals in which the sublimation enthalpy/lattice energy is determined by one type of intermolecular (non-covalent) interactions, such as CF_4_ [13,14]. A quantitative characteristic of intermolecular interactions in a crystal is the lattice energy or sublimation enthalpy, extrapolated to 0 K [15]. A number of theoretical approaches to the lattice energy assessment are reported in the literature. They mostly concern single-component crystals and use either quantum chemical modeling [16,17,18] or semi-empirical schemes [19,20,21]. In most of the papers cited above, short (strong) intermolecular H-bonds [22] are not considered since they are practically not realized in single-component crystals. On the other hand, such H-bonds determine the structure of multicomponent molecular crystals used in pharmaceuticals [23,24,25] and crystal engineering [26]. Intermolecular H-bonds of different types and strengths play a decisive role in the structure of crystal hydrates [27,28] and peroxosolvates [29,30].

Currently, there is a great demand for approaches that make it possible to quantitatively describe various intermolecular (non-covalent) interactions in organic crystals. In most cases, empirical approaches are used that relate the energy of the intermolecular interaction with one or another parameter of the electron density at the bond critical point [31]. In this case, the calculated values of the electron density, the values of the parameters retrieved from the precise X-ray diffraction data, and hybrid approaches are used [32]. The above approaches are often used to evaluate intermolecular interactions of different natures in various crystals [33,34,35], which gives rise to well-founded criticism [36,37]. Possible reasons for this criticism include the following. (1) The identification of a non-covalent interaction is based solely on the presence of the bond critical point [38]. (2) The limits of applicability of these approaches are not clear. For example, the approach developed in ref. [31] was used to estimate energies from ~1.5 [33] to ~600 kJ/mol [39]. (3) The dependence of the theoretical values of the electron density parameters at the bond critical point on the approximations used in the calculations by the DFT method has not been studied.

Peroxosolvates of organic molecules are convenient objects for a critical comparison of various DFT functionals and empirical approaches to assessing the energy of intermolecular interactions, that is, conventional H-bonds. At first, identification of the conventional H-bonds in crystals may be made without Bader analysis of periodic electron density [40]. Secondly, the enthalpy of H-bonds can be estimated by methods that do not use electron density characteristics, namely, changes in IR intensities [41], frequency shifts [40], and metric parameters of intermolecular H-bonds [42]. Third, organic crystals imply calculations with B3LYP and PBE-D3 functionals, which are widely used in calculations for periodic [12,14,17,18,34,43] and non-periodic [35] structures. Finally, H-bonds of various nature and strengths are realized in peroxosolvates [30].

**Scheme 1:**
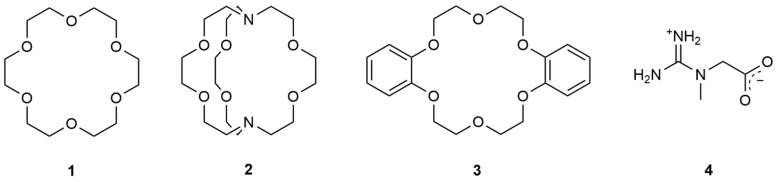
Structures of the macrocyclic ethers (**1**–**3**) and creatine (**4**), the peroxosolvates **I**–**IV** of which were obtained and studied by X-ray diffraction.

In the present paper, we focus on the applicability of the B3LYP and PBE-D3 functionals for evaluating the geometric parameters of intermolecular H-bonds of different types and strengths in peroxosolvates of macrocyclic ethers and amino acids (Scheme 1). Four of these two-component crystals were obtained and structurally characterized for the first time. A critical comparison of several methods for estimating the energy of intermolecular H-bonds is carried out, and various sources of errors are clarified. Finally, a fast quantum approach for evaluating the energy of non-covalent interactions in molecular crystals is suggested. This approach is based on non-periodic (cluster) models. Its accuracy and limits of applicability are demonstrated.

## 2. Results and Discussion

### 2.1. Crystal Structures and Hydrogen Bonding

**Figure 1:**
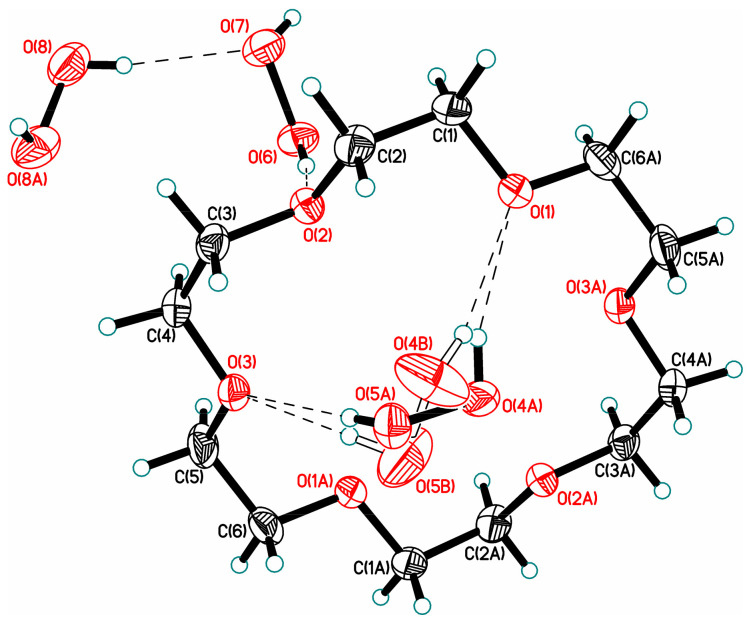
Asymmetric unit of **I**. Minor components of disorder are shown by open lines. Displacement ellipsoids are shown at the 50% probability level. The H-bonds are given by the dashed lines.

The structure of 18-crown-6 peroxosolvate C_12_H_24_O_6_•5H_2_O_2_ (**I**) comprises one independent cyclic ether molecule lying on a crystallographic inversion center and three solvent peroxide molecules (Figure 1). Two of them occupy general positions, and the third, namely H8-O8-O8A-H8A, is sited on a crystallographic two-fold axis. The molecule H4-O4-O5-H5 is disordered over two sites with an occupancy ratio of 0.927(3)/0.083(3) (Figure 1). In **I**, the ether molecule adopts an ordinary crown conformation with O1, O3, and O2A atoms lying on one side of the molecule and O2, O1A, and O3A on the other. The C–O bond lengths vary within the normal range (1.4209(12)–1.4288(11) Å) for 18-crown-6 derivatives (CSD ver. 5.43 November 2021 [44]). All oxygen atoms of organic coformer are involved in one hydrogen bond with neighboring H_2_O_2_ molecules. In the solvent molecules, O-O distances lie within the normal range for peroxosolvates (1.441(10)–1.4692(10) Å) [45]. The torsion H–O–O–H angles (80.8(18)–139.4(15)°) are also typical for solvent peroxide molecules.

**Figure 2:**
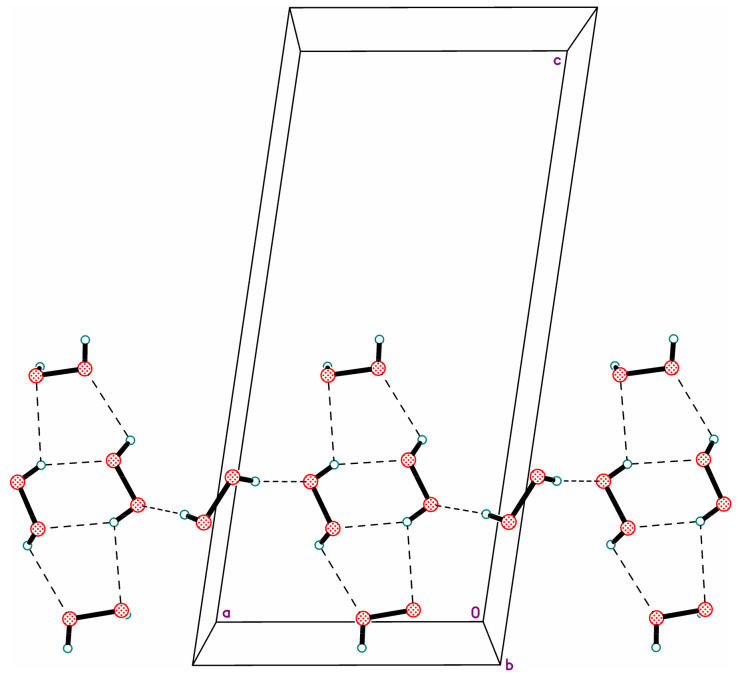
Infinite H-bonded peroxide chains in the structure **I**. Minor parts of disorder are not shown. Hydrogen bonds are indicated to be dashed lines.

As in the structures of all known peroxosolvates, H_2_O_2_ molecules in **I** participate in two donor hydrogen bonds [30]. Additionally, two of three peroxide molecules are involved in acceptor H-bonding. In the case of H6-O6-O7-H7 molecules, this hydrogen bonding results in the unprecedented formation of 2-fold cyclic dimers with R_2_^2^(6) topology (Figure S1). Of interest, atom H7 is involved in bifurcated H-bonding, and **I** represents the second example of a structure containing an H_2_O_2_ molecule with a bifurcated donor hydrogen bond [28]. Recently, it was supposed that the usage of organic coformers without donor H-atoms results in the targeted formation of peroxosolvates with infinite 1D-hydrogen peroxide clusters [46,47]. The structure of **I** is direct evidence of this rule (Figure 2). Within the chains, central network-forming H_2_O_2_ molecules are hydrogen bonded only with adjacent peroxides, while lateral molecules are additionally linked to crown ether coformers by donor HOOH∙∙∙OR_2_ bonds. To the best of our knowledge, this untrivial chain topology was not observed previously either for peroxosolvates or for hydrate structures.

**Figure 3:**
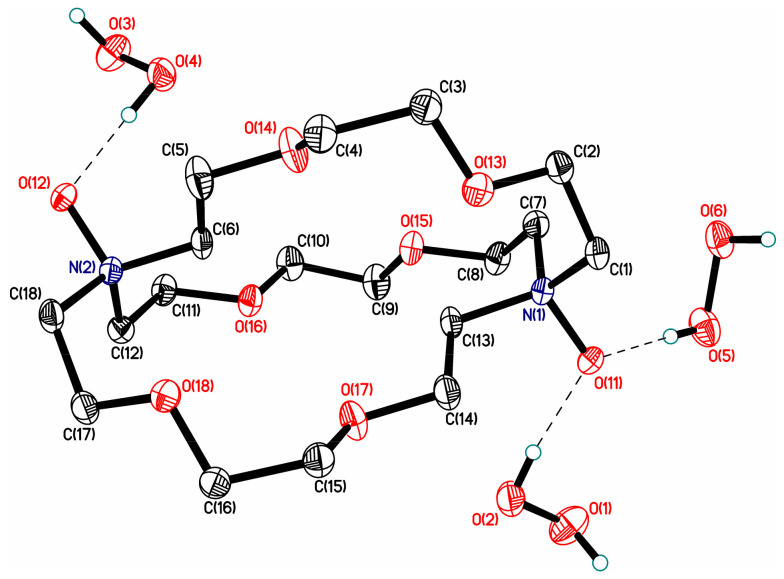
Asymmetric unit of **II**. Displacement ellipsoids are shown at the 50% probability level. The H-bonds are given by the dashed lines.

Trialkyl amines easily oxidize by hydrogen peroxide forming *N*-oxides [48]. We found that [2.2.2]-cryptand follows this rule and transforms to di-*N*-oxide in H_2_O_2_ solutions. The asymmetric unit in the structure of [2.2.2]-cryptand di-*N*-oxide peroxosolvate C_18_H_36_N_2_O_8_•3H_2_O_2_ (**II**) contains one cryptand coformer and three peroxide molecules (Figure 3). All molecules lie on general positions. In the organic molecule, all bond lengths and angles have ordinary values [44]. The coformer adopts “OO” conformation [49] with all ether oxygen atoms directed toward the center of the molecule. Obviously, this compact conformation prevents ether oxygen atoms from hydrogen bonding and denies the inclusion of solvents inside the cryptand skeleton. All solvent peroxide molecules possess skew conformation (85(2)-96(2)°) with O-O bond lengths varying within 1.4561(19)-1.4675(18) Å. All H_2_O_2_ molecules participate only in two approximately linear (170(2)-178(2)°) donor hydrogen bonds HOOH∙∙∙^−^O-N^+^ with short O∙∙∙O separations (2.6669(17)-2.7732(16) Å). Of interest, other possible H-bonds (HOOH∙∙∙OR_2_ or intersolvent HOOH∙∙∙O_2_H_2_) are not found in the structure. Probably, the latter results from the fact that charge-assisted HOOH∙∙∙^−^O-N^+^ hydrogen bonds are energetically preferable than all other possible HOOH∙∙∙O interactions in **II**. Of interest, both *N*-oxide oxygen atoms are involved in three hydrogen bonds with N^+^-O∙∙∙H angles ranging within 97.2–123.3°.

**Figure 4:**
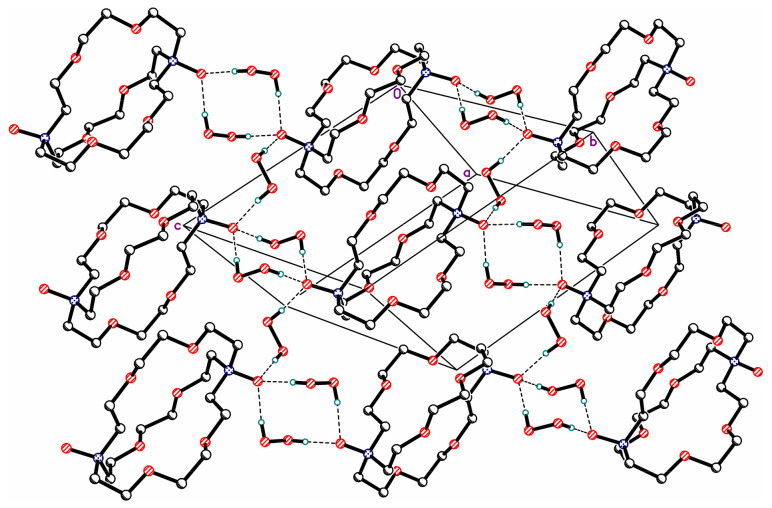
Hydrogen bonded layer in **II**. Hydrogen bonds are shown by dashed lines.

**Figure 5:**
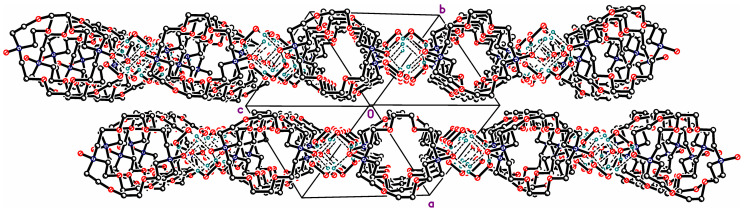
Hydrogen bonded layers in **II**. View along *abc*-diagonal.

In the crystal, the neighboring molecules are combined in N^+^-O^−^∙∙∙(HOOH)*n*∙∙∙^−^O-N^+^ (*n* = 1, 2) synthons. These fragments were shown to be structure-forming in peroxosolvates of organic *N*-oxides [50]. Finally, H-bonded layers perpendicular to *ab*-diagonal are formed in the structure **II** (Figure 4; Figure 5). To the best of our knowledge, **II** is the second example of a crystal structure containing [2.2.2]-cryptand di-*N*-oxide molecule [51].

**Figure 6:**
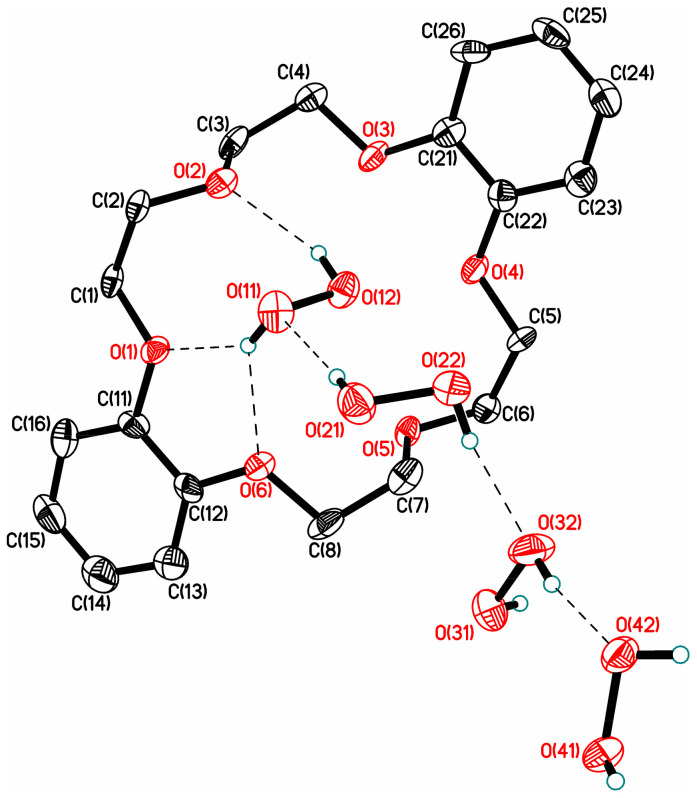
Asymmetric unit of **III**. Displacement ellipsoids are shown at the 50% probability level. The H-bonds are given by the dashed lines.

**Figure 7:**
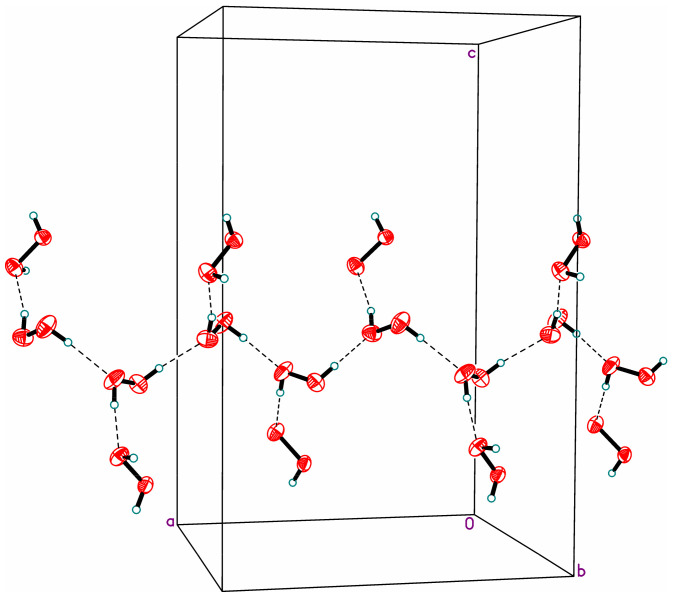
Hydrogen bonded chains along *a*-axis in **III**. Hydrogen bonds are shown by dashed lines.

The structure of dibenzo-18-crown-6 (db18c6) peroxosolvate C_20_H_24_O_6_•4H_2_O_2_ (**III**) comprises one independent cyclic ether molecule and four solvent peroxide molecules. All molecules lie on general positions (Figure 6). The metric parameters of the macrocyclic ether are ordinary with bond lengths close to those found in **I**. The organic coformer adopts an unusual “planar” conformation with approximately parallel aromatic rings (dihedral angle 1.5(4)°) and with both alkyl oxygen atoms O2 and O5 deviating in opposite directions from the mean plane of the molecule. In contrast, db18c6 possesses a bent conformation in the unsolvated structure [52], which coincides with the most stable ground state conformation in the gaseous phase [53]. According to CSD, a bent conformation with a dihedral angle between the aromatics of greater than 30° is also preferable for metal and H-bonded complexes of db18c6 (290 of total 298 entries). The remaining eight entries (BOYVED, DIHZEA, NOKVIU, OYAHAZ02, RAXJAB, RORXOL, VADTEZ, and XIGPEJ) correspond to planar conformation with a dihedral angle less than 5°, most of them possessing a crystallographically imposed central symmetry. Five ether oxygen atoms participate in hydrogen bonds with hydrogen peroxide. Only catecholate atom O3 is not involved in H-bonding. The peroxide molecules possess skew conformation (77(7)-117(7)°) with O-O bond lengths ranging from 1.427(6) to 1.465(6) Å. All of them participate in two donor hydrogen bonds and one inter-solvent acceptor HOOH∙∙∙O_2_H_2_ linkage. As expected, the latter results in formation of hydrogen-bonded peroxide chains with only lateral peroxide molecules H11-O11-O12-H12 and H41-O41-O42-H42 interacting with the organic coformer (Figure 7).

**Figure 8:**
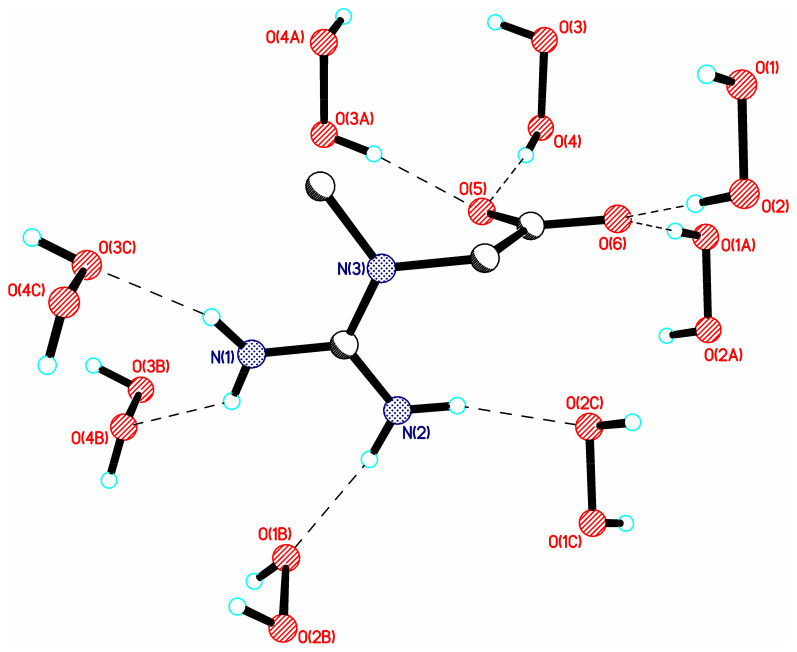
Hydrogen bonding of creatine in **IV**. Hydrogen bonds are shown by dashed lines.

The asymmetric unit in the structure of creatine peroxosolvate C_4_H_9_N_3_O_2_•2H_2_O_2_ (**IV**) contains one independent zwitterionic molecule of natural amino acid and two H_2_O_2_ molecules (Figure 8). All geometric parameters of the organic molecule (including conformation) are close to those previously found for the structures of creatine monohydrate and three polymorphs of unsolvated creatine [54,55,56].

In these four structures, the characteristic torsion angle C-N(Me)-CH_2_-C between two planar fragments (guanidinium and amino acid) varies from 68 to 76°, while in **IV**, this angle is equal to 81.9(1)°. In **IV**, all creatine hydrogen atoms are involved in H-bonding (Figure 8). Of interest, all four *sp*^2^-hybridized lone electron pairs of the carboxylate group participate in hydrogen bonds with neighboring peroxide molecules resulting in [SA; SA] linkage mode [57].

**Figure 9:**
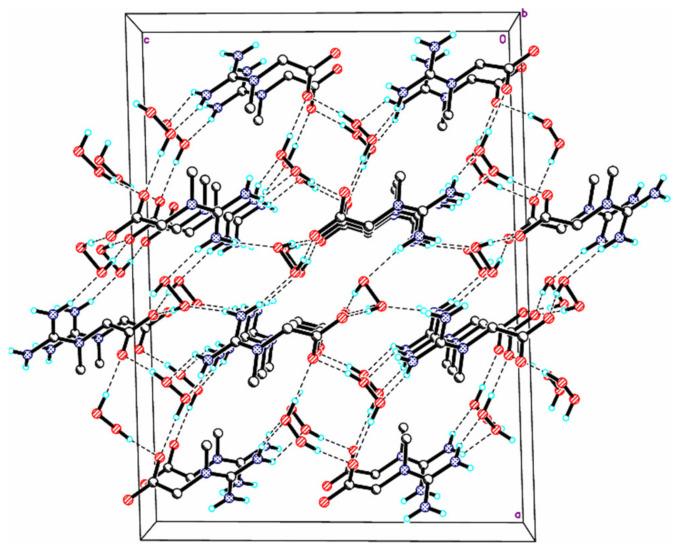
Three-dimensional packing motif in **IV**. Hydrogen bonds are shown by dashed lines.

H_2_O_2_ molecules adopt skew geometry (96.4(15) and 96.9(16)°) with O-O bond lengths equal to 1.4602(11) and 1.4643(10) Å. They both are involved in two donor HOOH∙∙∙^−^O_2_C- and two acceptor NH∙∙∙O_2_H_2_ hydrogen bonds. In the crystal, creatine and peroxide molecules are combined in a complex 3D hydrogen-bonded motif (Figure 9).

The crystal structure of phenylserine peroxosolvate (**V**) (Figure S5) was discussed in detail previously [58].

### 2.2. H-Bond Enthalpies/Energies in Peroxosolvates of Macrocyclic Ethers and Amino Acids

It was noted that there are several empirical approaches to assessing the enthalpy Δ*H_HB_* of the intermolecular H-bond in a solid. The most accurate of these is Iogansen’s approach to exploring changes in IR intensities [41]. In contrast to the liquid state [59], the experimental determination of the integral intensities of OH stretching vibrations in crystals is a non-trivial problem. Periodic DFT calculations of IR intensities are usually performed in the “double harmonic approximation” [60]. This has limited applicability for estimating the frequencies and IR intensities of stretching vibrations of OH/NH groups involved in the formation of short (strong) H-bonds due to significant mechanical and electro-optical anharmonicity. A theoretical description of the considered bands beyond the “double harmonic approximation” is a time-consuming procedure [61,62,63]. On the other hand, the Iogansen approach exploring changes in OH stretching frequencies to estimate Δ*H_HB_* has limited applicability to ionic H-bonds [30] since some ^+^N-H∙∙∙O^−^ bonds are not realized in the gas phase. Moreover, the application of this approach to bifurcated H-bonds is not obvious. This is why we use the approaches described below.

The Δ*H_HB_* values are estimated using the Rozenberg approach [42]:

−Δ*H_HB_* [kJ/mol] = 0.134·*R*(H∙∙∙O)^−3.050^ (1)

where the *R*(H∙∙∙O) is the H∙∙∙O distance (nm). Equation (1) describes −Δ*H_HB_* varying from 10 to 80 kJ/mol [42]. The *R*(H∙∙∙O) values are obtained as a result of geometry optimization.

The energy of intermolecular H-bonds *E_HB_* in the considered crystals is evaluated according to ref. [64] as:

E_HB_ [kJ/mol] = 1124·*G_b_* [atomic units] (2)

where *G_b_* is the positively-defined local electronic kinetic energy density at the H∙∙∙O bond critical point [38]. Equation (2) will be referred to as the *G_b_*–*E_HB_* approach below.

We start with crystal **I** (crown Figure S1) since it has weak H-bonds (−Δ*H_HB_* < 25 kJ/mol, *R*(H∙∙∙O) > 1.80 Å and *ρ*_b_ < 0.04 a.u.). Approaches (1) and (2) lead to similar results for these H-bonds (Table S1a). Moderate H-bonds (25 < −Δ*H_HB_* < 50 kJ/mol, 1.80 > *R*(H∙∙∙O) > 1.60 Å and 0.04 < *ρ*_b_ < 0.06 a.u.) exist in crystalline **II** (cryptand), **III** (benzocrown), and creatine diperoxosolvate **IV**, (Figures S2–S4). The differences between the two approaches can reach 10 kJ/mol (Tables S2a–S4a), and the absolute enthalpy values always turn out to be lower than the calculated values of the energy of the moderate H-bonds. The obtained results agree with the literature data [65,66,67,68]. 

For short (strong) H-bonds (−Δ*H_HB_* > 50 kJ/mol, *R*(H∙∙∙O) < 1.60 Å and *ρ*_b_ > 0.60 a.u.) which exist in crystalline 3-phenylserine peroxosolvate **V** [69] (refcode VILGAB, Figure S5) the differences between −Δ*H_HB_* and *E_HB_* can be 15 kJ/mol (Table S5a) or more (Table 2 in [65], Table 1 in [68]). 

**Figure 10:**
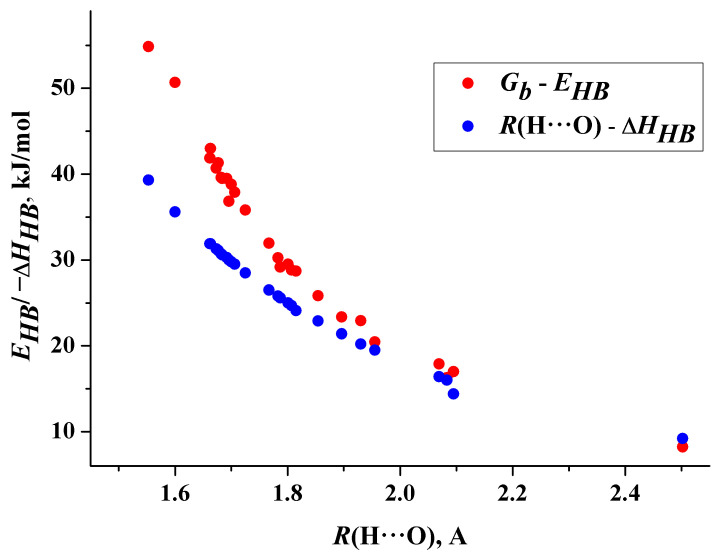
The H-bond enthalpies, −Δ*H_HB_* (Equation (1), blue circles) and the energies, *E_HB_* (Equation (2), red circles), evaluated for the considered molecular crystals versus the H∙∙∙O distances computed at the B3LYP/6-31G** level.

The −Δ*H_HB_* and *E_HB_* values evaluated for the considered molecular crystals versus the calculated H∙∙∙O distances are given in Figure 10. With a decrease in the H∙∙∙O distance (that is, an increase in the strength of H-bonds), the difference between the energy values calculated using Equations (1) and (2) increases. Since the accuracy of the Rozenberg approach for the considered H-bonds is several kJ/mol, we conclude that Equation (2) can significantly overestimate *E_HB_* for short (strong) H-bonds in molecular crystals. 

The specific feature of Equations (1) and (2) is the possibility of using the *G_b_* and *R*(H∙∙∙O) values obtained from experiments (high precision X-ray diffraction, neutron diffraction) or the periodic DFT computations followed by the Bader analysis of the periodic electronic density. From a theoretical point of view, Equation (1) presents the simplest case because the *R*(H∙∙∙O) values are obtained as a result of geometry optimization. From the experimental point of view, the applicability of this approach is not straightforward due to the usual lack of accuracy that X-ray structures have on the H atoms. It should be noted that neutron diffraction data on H-bonded crystals are very limited [69]. Initially, Equation (2) was developed for gas-phase systems with intermolecular H-bonds with energies ranging from 3 to 90 kJ/mol [31,64]. Currently, approaches that use the characteristics of the electron density at the point (3, −1) are used to estimate the energies of non-covalent interactions in various systems in gas and condensed media, and the *E_HB_* values vary within wide limits [33,39]. From a theoretical point of view, errors in the energy values obtained using Equation (2) arise due to inaccuracies in the calculated values of the metric parameters of H-bonds and the use of non-periodic models in the computations. From an experimental point of view, errors in the *E_HB_* values are due to the use of the Kirzhnitz approximation [70].

To estimate the error in determining the −Δ*H_HB_* value caused by the use of B3LYP, the *R*(H∙∙∙O) values were computed at the PBE-D3/6-31G** level (Tables S6–S8). The −Δ*H_HB_* values calculated using PBE-D3 agree nicely with those obtained using B3LYP. In accordance with the literature ([28] and Table S1a in [71]), the PBE-D3 calculations slightly overestimate the H-bonded energy compared to the B3LYP calculations.

### 2.3. Fast Quantum Approach for Evaluating the Energy of Intermolecular H-Bonds in Molecular Crystals

The cluster approximation is widely used to describe the structure and properties of organic crystals: the energy of the intermolecular interactions [72,73], the mobility of charges in organic semiconductors [74,75], and the chemical shift of the nucleus [76]. This requires knowledge of the structure of the considered crystal, that is, the cif file. Usually, a cluster consisting of several molecules is cut out of a crystal, and the geometry is partially optimized, i.e., the position of only hydrogen atoms is varied [77]. In contrast to C-H bonds whose length can be uniformly normalized [78], the length of OH and NH depends on the strength of the H-bonds formed by these groups (Table 2 in [22]). Note that the complete optimization of the cluster under non-periodic conditions can significantly change the mutual orientation of the molecules, that is, the number and strength of the studied H-bonds [79].

The cluster size is determined by the condition that all “unique” H-bonds that occur in the crystal under study are taken into account. In some cases, it can be quite large. For all the crystals considered, except for crystal III, the cluster includes no more than 100 atoms. Calculations of such clusters are an order of magnitude faster than calculations of crystals (geometry optimization). The total enthalpies/energies of all unique H-bonds in each of the studied crystals, calculated using periodic DFT, are given in Tables SXa in Supplementary Materials, where X = 1, 2, … 5. The corresponding values obtained in the cluster approximation are given in Tables SXb in Supplementary Materials, where X = 1, 2, … 5. It follows that the total enthalpies/energies calculated using the cluster approximation are in good agreement with the values obtained from periodic DFT calculations. This justifies the use of the cluster approximation for *estimating* the energies of H-bonds in molecular crystals. The *G_b_*-*E_HB_* approach gives reasonable results for energies from 2.0 [79] to 50 kJ/mol and is applicable to describe bifurcated H-bonds formed by the OH group. This has been called the fast quantum approach.

It should be emphasized that the analysis of normal vibrations of the cluster structure, obtained as a result of partial optimization usually leads to imaginary frequencies. Therefore, this approximation is hardly applicable for calculating the experimentally observed characteristics of molecular crystals, namely: sublimation enthalpies [17,18,43], IR [80,81] and Raman spectra [68,71].

Unlike Equation (1), the *G_b_*-*E_HB_* approach can be applied to intramolecular H-bonds [82,83], which play a structure-directed role in pharmaceutical multicomponent organic crystals [84,85]. It should be noted that empirical approaches use different geometric parameters to estimate the energies of inter- and intramolecular H-bonds in organic crystals, compare [42] with [86].

## 3. Materials and Methods

### 3.1. Compounds and Solvents

Macrocyclic ethers (dibenzo-18-crown-6, 18-crown-6, [2.2.2]cryptand), creatine, and 50% hydrogen peroxide were purchased from Sigma-Aldrich (St. Louis, MO, USA), Protein Company (Moscow, Russia), and Fisher Scientific (Waltham, MA, USA), respectively. In total, 98% hydrogen peroxide was prepared by an extraction method [87]. Handling procedures for concentrated hydrogen peroxide are described in detail (danger of explosion!) [88,89].

### 3.2. Cocrystal Preparation

Colorless crystals of **I**–**IV** were obtained by cooling to −21 °C saturated solutions (rt) of respective compounds in 98% hydrogen peroxide. Compounds **I** and **III** are stable just for a few minutes without mother liquor, while crystal **IV** is stable for several hours. In contrast, the peroxosolvate of cryptand **II** demonstrates high stability and does not exhibit noticeable decay for several months.

### 3.3. Single Crystal X-ray Diffraction Experiments

The crystals were extracted from the mother liquor and covered immediately with inert oil to prevent contact with atmospheric moisture. Experimental data sets were collected on a Bruker SMART APEX II diffractometer (for **I**, **III**, and **IV**) and a Bruker SMART Photon II diffractometer (for **II**). Absorption corrections based on measurements of equivalent reflections were applied [90]. The structures were solved by direct methods and refined by full-matrix least-squares on *F*^2^ with anisotropic thermal parameters for all non-hydrogen atoms [91]. The studied thin plate crystal of **II** was found to be twinned by the law (1–10) [5,6,7,8,9,10,11,12,13,14,15,16,17,18,19,20,21,22,23,24,25,26,27,28,29,30,31] with an approximate domain ratio of 0.79/0.21. Both domains were indexed separately, and full data were integrated and scaled for absorption with two matrices (APEX III software). The data for the main domain were used for calculations. In **I**, one of the peroxide molecules was disordered over two sites with an occupancy ratio of 0.927(3)/0.083(3). All hydrogen atoms in **I** and **IV** were found from difference Fourier synthesis and refined with isotropic thermal parameters (except for a minor part of the disordered H_2_O_2_ molecule). In **II** and **III**, all carbon H atoms were placed in calculated positions and refined using a riding model, while “active” hydrogen atoms were found from difference Fourier synthesis, and their positional parameters were refined. In all structures, the partial substitutional disorder of hydrogen peroxide by water molecules [92,93,94,95] was not observed since no residual peaks with an intensity more than 0.17 e/A^3^ were seen in the hydrogen peroxide molecule regions. Crystal data and details of X-ray analysis are given in Table S5a. CCDC 2158207-2158210 contains the supplementary crystallographic data for **I**–**IV**. These data can be obtained free of charge via http://www.ccdc.cam.ac.uk/conts/retrieving.html (accessed on 18 May 2022) (or from the CCDC, 12 Union Road, Cambridge CB2 1EZ, UK; Fax: +44-1223-336033; E-mail: deposit@ccdc.cam.ac.uk).

### 3.4. Periodic (Solid-State) DFT Computations

B3LYP and PBE-D3 are the most popular functionals in periodic (solid-state) DFT calculations of organic crystals [12,17,18,81]. These functionals provide a grounded trade-off between the accuracy and the rate of calculations of experimentally observed properties of multicomponent organic crystals [28,43,68,71]. Computations with all-electron Gaussian-type localized orbital basis 6-31G** were conducted using the CRYSTAL17 package [96]. We employed B3LYP [97,98] and PBE [99]. The London dispersion interactions were taken into account by introducing the D3 correction with Becke–Jones damping (PBE-D3) developed by Grimme et al. [100]. The space groups and the unit cell parameters of the considered two-component crystals (**I**, **II**, **III**, **IV**, and **V**) obtained from the X-ray diffraction experiment were fixed, and the structural relaxations were limited to the positional parameters of the atoms (AtomOnly). The model structure of **I** was obtained from the crystal structure, which has one crystallographically independent hydrogen peroxide molecule disordered over two sites with an occupancy ratio of 0.927(3)/0.073(3). Only the higher occupancy was considered in our computations. Tolerance on energy controlling the self-consistent field convergence for geometry optimizations and frequencies computations is set to 10^−10^ and 10^−11^ hartree, respectively. The shrinking factor of the reciprocal space net is set to 3. All structures optimized at the B3LYP/6-31G** level are found to correspond to the minimum point on the potential energy surface. Further details of calculations are given in the Supplementary Materials.

## 4. Conclusions

Four new crystalline peroxosolvates of 18-crown-6, [2.2.2]-cryptand, dibenzo-18-crown-6, and creatine were crystallized from the solution of the corresponding coformer in 98% hydrogen peroxide. H_2_O_2_ participate in the formation of moderate hydrogen bonds with organic coformer and adjacent molecules of hydrogen peroxide.

Hydrogen bond enthalpies/energies in peroxosolvates of macrocyclic ethers and amino acids are evaluated using the approaches based on the H∙∙∙O distance (the *R*(H∙∙∙O)-Δ*H_HB_* approach) and the local electronic kinetic *G_b_* densities at the hydrogen bond critical point (the *G_b_*-*E_HB_* approach). In the case of weak and moderate hydrogen bonds, both approaches give similar results. For short (strong) hydrogen bonds (−Δ*H_HB_* > 50 kJ/mol, *R*(H∙∙∙O) < 1.60 Å and *ρ*_b_ > 0.60 a.u.), the differences −Δ*H_HB_* and *E_HB_* can be 15 kJ/mol or more. We conclude that the *G_b_*-*E_HB_* approach should be used with caution for describing short (strong) hydrogen bonds in organic crystals. 

Reasonable agreement between the hydrogen bonds enthalpies/energies obtained using the H∙∙∙O distances and the local electronic kinetic *G_b_* densities at the hydrogen bond critical point in crystalline peroxosolvates indicates that the use of the Bader approach for localization and characterization of intermolecular hydrogen bonds in organic crystals is fully justified.

It is shown that the cluster approximation can be used to *evaluate* intermolecular interactions using the *R*(H∙∙∙O)-Δ*H_HB_* and *G_b_*-*E_HB_* approaches. The latter approach gives reasonable results for energies from 2.0 to 50 kJ/mol and is applicable to describe bifurcated hydrogen bonds formed by the OH group. It has been called the fast quantum approach. Due to the use of partial optimization, the cluster approximation is hardly applicable for calculating sublimation enthalpies, IR and Raman spectra, and thermodynamic and elastic properties of molecular crystals.

## Data Availability

Supplementary crystallographic data for **I**–**IV** can be obtained free of charge via http://www.ccdc.cam.ac.uk/conts/retrieving.html (accessed on 18 May 2022). The data presented in this study are available on request from the corresponding author.

## Supplementary Materials

The following supporting information can be downloaded at: https://www.mdpi.com/article/10.3390/molecules27134082/s1, References [38,75,101,102,103,104,105] are cited in Supplementary Materials; Section S1, Details of periodic (solid-state) DFT computations; Section S2, Non-periodic DFT computations; Figure S1: H-bonding network of H_2_O_2_ molecules in the crystal structure of **I**. Peroxide molecule O(4B)-O(5B) represents minor part of disorder. Displacement ellipsoids are shown at the 50% probability level. The H-bonds are given by the dashed lines. Symmetry operation: (i) 1 − x, 1 − y, 1 − z; (ii) 2 − x, y, 1.5 − z; (iii) 1 − x, y, 1.5 − z; (iv) x, 1 − y, 0.5 + z.; Figure S2: Part of the H-bond network in **II**. H atoms omitted for clarity. Displacement ellipsoids are shown at the 50% probability level. The H-bonds are given by the dashed lines. Symmetry operation: (i) 1 − x, 2 − y, 1 − z; (ii) −x, 1 − y, 2 − z; (iii) x, y, 1 + z; Figure S3: Part of the H-bond network in **III**. Displacement ellipsoids are shown at the 50% probability level. H atoms of macrocyclic ether are omitted for clarity. The H-bonds are given by the dashed lines. Symmetry operation: (i) −0.5 + x, 1.5 − y, z; (ii) 0.5 − x, 0.5 + y, −0.5 + z.; Figure S4; Part of the H-bond network in creatine peroxosolvate **IV**. Displacement ellipsoids are shown at the 50% probability level. H atoms of macrocyclic ether omitted for clarity. The H-bonds are given by the dashed lines. Symmetry operation: (i) x, 1 − y, −0.5 + z; (ii) x, −1 + y, z; (iii) 0.5 − x, 1.5 − y, 1 − z; (iv) 1 − x, 1 − y, 1 − z; Figure S5: Part of the H-bond network in 3-phenylserineperoxosolvate **V**. Displacement ellipsoids are shown at the 50% probability level. Only «active» hydrogen atoms are shown. The H-bonds are given by the dashed lines. Symmetry operation: (i) −x, y, −z; (ii) 0.5 − x, 0.5 − y, 0.5 + z; (iii) x, y, −1 + z; Table S1a: B3LYP/6-31G** values of the (H∙∙∙O) distance, *R*(H∙∙∙O), the electron density, *ρ*_b_, and the local electronic kinetic energy density, *G_b_*, at the H∙∙∙O bond critical point in **I**. The H-bond energy *E_HB_* and enthalpy Δ*H_HB_* evaluated using Equations (2) and (1), respectively, are given in the last two columns; Table S1b: The values of (H∙∙∙O) distance, *R*(H∙∙∙O), the electron density, *ρ*_b_, and the local electronic kinetic energy density, *G_b_*, at the O∙∙∙O bond critical point obtained using partial B3LYP/6-31G** optimization of the **I** cluster. The H-bond energy *E_HB_* and enthalpy Δ*H_HB_* evaluated using Equations (2) and (1), respectively, are given in the last two columns.; Table S2a: B3LYP/6-31G** values of the electron density, *ρ*_b_, and the local electronic kinetic energy density, *G_b_,* at the H∙∙∙O bond critical point in **II**. The H-bond energy *E_HB_* and enthalpy Δ*H_HB_* evaluated using Equations (2) and (1), respectively, are given in the last two columns.; Table S2b: The values of (H∙∙∙O) distance, *R*(H∙∙∙O), the electron density, *ρ*_b_, and the local electronic kinetic energy density, *G_b_,* at the O∙∙∙O bond critical point obtained using partial B3LYP/6-31G** optimization of the **II** cluster. The H-bond energy *E_HB_* and enthalpy Δ*H_HB_* evaluated using Equations (2) and (1), respectively, are given in the last two columns; Table S3a; B3LYP/6-31G** values of the (H∙∙∙O) distance, *R*(H∙∙∙O), the electron density, *ρ*_b_, and the local electronic kinetic energy density, *G_b_,* at the O∙∙∙O bond critical point in **III**. The H-bond energy *E_HB_* and enthalpy Δ*H_HB_* evaluated using Equations (2) and (1), respectively, are given in the last two columns; Table S3b: The values of (H∙∙∙O) distance, *R*(H∙∙∙O), the electron density, *ρ*_b_, and the local electronic kinetic energy density, *G_b_,* at the O∙∙∙O bond critical point obtained using partial B3LYP/6-31G** optimization of the **III** cluster. The H-bond energy *E_HB_* and enthalpy Δ*H_HB_* evaluated using Equations (2) and (1), respectively, are given in the last two columns; Table S4a; B3LYP/6-31G** values of the (H∙∙∙O) distance, *R*(H∙∙∙O), the electron density, *ρ*_b_, and the local electronic kinetic energy density, *G_b_,* at the O∙∙∙O bond critical point in creatine peroxosolvate **IV**. The H-bond energy *E_HB_* and enthalpy Δ*H_HB_* evaluated using Equations (2) and (1), respectively, are given in the last two columns; Table S4b: The values of (H∙∙∙O) distance, *R*(H∙∙∙O), the electron density, *ρ*_b_, and the local electronic kinetic energy density, *G_b_,* at the O∙∙∙O bond critical point obtained using partial B3LYP/6-31G** optimization of the **IV** cluster. The H-bond energy *E_HB_* and enthalpy Δ*H_HB_* evaluated using Equations (2) and (1), respectively, are given in the last two columns; Table S5a: B3LYP/6-31G** values of the (H∙∙∙O) distance, *R*(H∙∙∙O), the electron density, *ρ*_b_, and the local electronic kinetic energy density, *G_b_,* at the O∙∙∙O bond critical point in 3-phenylserine peroxosolvate **V** (refcode VILGAB). The H-bond energy *E_HB_* and enthalpy Δ*H_HB_* evaluated using Equations (2) and (1), respectively, are given in the last two columns; Table S5b: The values of (H∙∙∙O) distance, *R*(H∙∙∙O), the electron density, *ρ*_b_, and the local electronic kinetic energy density, *G_b_,* at the O∙∙∙O bond critical point obtained using partial B3LYP/6-31G** optimization of the **V** cluster. The H-bond energy *E_HB_* and enthalpy Δ*H_HB_* evaluated using Equations (2) and (1), respectively, are given in the last two columns; Table S6: Distances between the atoms involved in the formation of intermolecular H-bonds *R*(O∙∙∙O) and *R*(H∙∙∙O) in **I**^a)^, obtained using periodic DFT computations (PBE-D3/6-31G** and B3LYP/6-31G**). Theoretical values of the enthalpy, Δ*H_HB_* of intermolecular H-bonds evaluated using Equation (1) are given in the last two columns; Table S7: Distances between the atoms involved in the formation of intermolecular H-bonds *R*(O∙∙∙O) and *R*(H∙∙∙O) in **II**, obtained using periodic DFT computations (PBE-D3/6-31G** and B3LYP/6-31G**). Theoretical values of the enthalpy, Δ*H_HB_* of intermolecular H-bonds evaluated using Equation (1) are given in the last two columns; Table S8: Distances between the atoms involved in the formation of intermolecular H-bonds *R*(X∙∙∙O) and *R*(H∙∙∙O) in creatine diperoxosolvate IV, where X = N or O, obtained using periodic DFT computations (PBE-D3/6-31G** and B3LYP/6-31G**). Theoretical values of the enthalpy, Δ*H_HB_* of intermolecular H-bonds evaluated using Equation (1) are given in the last two columns; Table S9: X-ray structure determination summary.

## Sample Availability

No possibility.
