# Supplementary material for: Fast Quantum Approach for Evaluating the Energy of Non-Covalent Interactions in Molecular Crystals: The Case Study of Intermolecular H-Bonds in Crystalline Peroxosolvates
Source: Molecules. 2022 Jun 24;27(13):4082. doi: 10.3390/molecules27134082 (PMC9268483; doi:10.3390/molecules27134082)
Supplement: Supplementary file 1 [file molecules-27-04082-s001.zip › molecules-1754863-supplementary final.pdf]

Supporting Information for publication

Fast Quantum Approach for Evaluating the Energy of Non-Covalent Interactions in Molecular Crystals: The Case Study of Intermolecular H-Bonds in Crystalline Peroxosolvates

Alexander G. Medvedev [1], Andrei V. Churakov [1], Mger A. Navasardyan [1], Petr V. Prikhodchenko [1], Ovadia Lev[2] and Mikhail V. Vener [1,*]

[1] Kurnakov Institute of General and Inorganic Chemistry, Russian Academy of Sciences, Leninskiy Prospekt 31, Moscow 119991, Russia; mag@igic.ras.ru (A.G.M.); churakov@igic.ras.ru (A.V.C.);
navasardyan@igic.ras.ru (M.A.N.); prikhman@gmail.com (P.V.P.)

[2] The Casali Center of Applied Chemistry, The Institute of Chemistry, The Hebrew University of Jerusalem, Jerusalem 9190401, Israel; ovadia@mail.huji.ac.il

* Correspondence: vener@igic.ras.ru

Section S1. Details of periodic (solid-state) DFT computations.

The B3LYP /6-31G** and PBE-D3/6-31G** optimized structures were used in computations of the periodic electronic wave-functions by CRYSTAL98 [101]. The quantum theory of atoms in molecules and crystals (Bader) analysis of the crystalline electron density [38] is performed with TOPOND [102]. The calculation methodology is presented elsewhere [103]. Imaginary frequencies are found for structures **I**, **III** and **V** optimized at the PBE-D3/6-31G** level. This problem is usually solved by reducing the space symmetry of the crystal [75]. Reducing the space symmetry group of the crystal **I** to P-1 and **V** to P-4 made it possible to get rid of imaginary frequencies.

Section S2. Non periodic DFT computations.

Non periodic DFT computations are performed with Gausiian16 [104]. The "opt=readopt" option were used. Bader analysis of the non periodic electron density is performed using AIMALL [105].

Table S1a. B3LYP/6-31G** values of the (H···O) distance, R(H···O), the electron density, ρ_b, and the local electronic kinetic energy density, G_b, at the H···O bond critical point in **I**. The H-bond energy E_{HB} and enthalpy ΔH_{HB} evaluated using Equations 2 and 1, respectively, are given in the last two columns.

H-bonded fragment[a]	R(H···O), Å	ρ_b (a.u.)	G_b (a.u.)	E_{HB}, (kJ mol^{-1})	$-\Delta H_{HB}$ (kJ mol^{-1})
O(4A)-H(4A)…O(1)	1.896	0.0278	0.0208	**23.4**	**21.4**
O(5A)-H(5A)…O(3)	1.807	0.0338	0.0256	**28.8**	**24.7**
O(6)-H(6)…O(2)	1.783	0.0370	0.0269	**30.3**	**25.8**
O(7)-H(7)…O(6)^{iii}	1.930	0.0275	0.0204	**22.9**	**20.2**
O(7)-H(7)…O(5A)^{iv}	2.502	0.0084	0.0073	**8.2**	**9.2**
O(8)-H(8)…O(7)	1.854	0.0316	0.0229	**25.8**	**22.9**
Σ				**139.4**	**124.2**

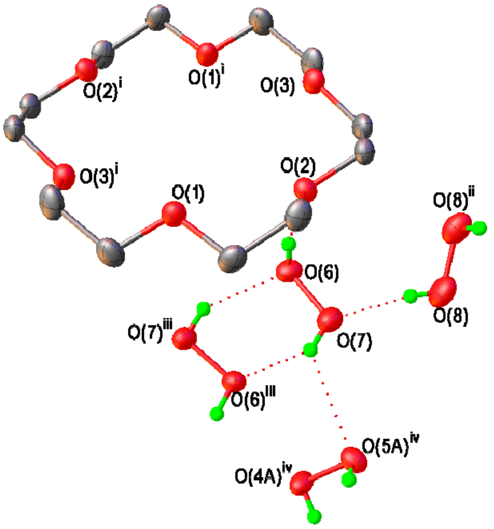

Figure S1. H-bonding network of H_2O_2 molecules in the crystal structure of **I**. Peroxide molecule O(4B)-O(5B) represents minor part of disorder. Displacement ellipsoids are shown at the 50% probability level. The H-bonds are given by the dashed lines. Symmetry operation: (i) 1-x,1-y,1-z; (ii) 2-x,y,1.5-z; (iii) 1-x,y,1.5-z; (iv) x,1-y,0.5+z.

Table S1b. The values of (H···O) distance, R(H···O), the electron density, ρ_b, and the local electronic kinetic energy density, G_b, at the O···O bond critical point obtained using partial B3LYP/6-31G** optimization of the **I** cluster. The H-bond energy E_{HB} and enthalpy ΔH_{HB} evaluated using Equations 2 and 1, respectively, are given in the last two columns.

H-bonded fragment[a]	R(H···O), Å	ρ_b (a.u.)	G_b (a.u.)	E_{HB}, (kJ mol^{-1})	$-\Delta H_{HB}$ (kJ mol^{-1})
O(4A)-H(4A)…O(1)	1.909	0.0273	0.0206	**23.2**	**20.9**
O(5A)-H(5A)…O(3)	1.860	0.0303	0.0230	**25.9**	**22.7**
O(6)-H(6)…O(2)	1.786	0.0372	0.0267	**30.0**	**25.6**
O(7)-H(7)…O(6)^{iii}	1.971	0.0258	0.0188	**21.1**	**19.0**
O(7)-H(7)…O(5A)^{iv}	2.381	0.0111	0.0090	**10.1**	**10.7**
O(8)-H(8)…O(7)	1.879	0.0302	0.0219	**24.6**	**22.0**
Σ				**134.9**	**120.9**

Table S2a. B3LYP/6-31G** values of the electron density, ρ_b, and the local electronic kinetic energy density, G_b, at the H···O bond critical point in **II**. The H-bond energy E_{HB} and enthalpy ΔH_{HB} evaluated using Equations 2 and 1, respectively, are given in the last two columns.

H-bonded fragment[a]	R(H···O), Å	ρ_b (a.u.)	G_b (a.u.)	E_{HB}, (kJ mol^{-1})	$-\Delta H_{HB}$ (kJ mol^{-1})
$O(1)^{i}$-$H(1)^{i}$…O(11)	1.677	0.0486	0.0367	**41.3**	**31.1**
O(2)-H(2)…O(11)	1.700	0.0465	0.0345	**38.8**	**29.8**
O(5)-H(5)…O(11)	1.663	0.0511	0.0382	**43.0**	**31.9**
O(4)-H(4)…O(12)	1.706	0.0449	0.0336	**37.9**	**29.5**
$O(3)^{ii}$-$H(3)^{ii}$…O(12)	1.725	0.0438	0.0318	**35.8**	**28.5**
$O(6)^{iii}$-$H(6)^{iii}$…O(12)	1.767	0.0396	0.0284	**31.9**	**26.5**
Σ				**228.7**	**177.5**

[a] See **Figure S2**.

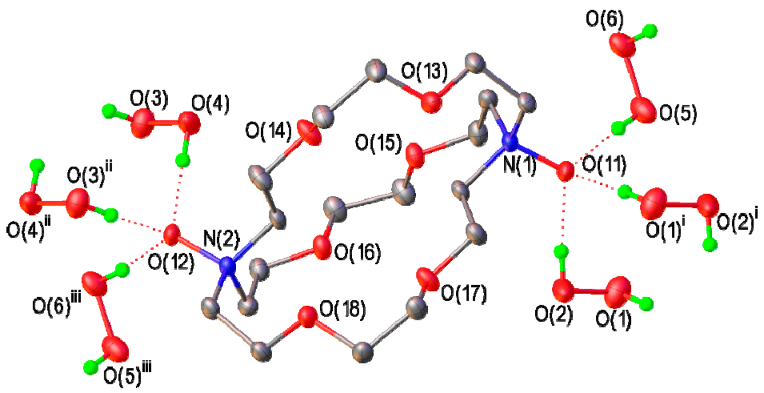

Figure S2. Part of the H-bond network in **II**. H atoms omitted for clarity. Displacement ellipsoids are shown at the 50% probability level. The H-bonds are given by the dashed lines. Symmetry operation: (i) 1-x,2-y,1-z; (ii) -x,1-y,2-z; (iii) x,y,1+z.

Table S2b. The values of (H···O) distance, *R*(H···O), the electron density, ρ_b, and the local electronic kinetic energy density, G_b, at the O···O bond critical point obtained using partial B3LYP/6-31G** optimization of the **II** cluster. The H-bond energy E_{HB} and enthalpy ΔH_{HB} evaluated using Equations 2 and 1, respectively, are given in the last two columns.

H-bonded fragment[a]	*R*(H···O), Å	ρ_b (a.u.)	G_b (a.u.)	E_{HB}, (kJ mol^{-1})	$-\Delta H_{HB}$ (kJ mol^{-1})
O(1)^{i}-H(1)^{i}…O(11)	1.708	0.0451	0.0337	**37.9**	**29.4**
O(2)-H(2)…O(11)	1.695	0.0473	0.0362	**40.7**	**30.1**
O(5)-H(5)…O(11)	1.776	0.0389	0.0281	**31.6**	**26.1**
O(4)-H(4)…O(12)	1.678	0.0490	0.0369	**41.5**	**31.0**
O(3)^{ii}-H(3)^{ii}…O(12)	1.734	0.0425	0.0319	**35.9**	**28.1**
O(6)^{iii}-H(6)^{iii}…O(12)	1.798	0.0370	0.0267	**30.0**	**25.1**
Σ				**217.5**	**169.8**

[a] See Figure S2

Table S3a. B3LYP/6-31G** values of the (H···O) distance, R(H···O), the electron density, ρ_b, and the local electronic kinetic energy density, G_b, at the O···O bond critical point in **III**. The H-bond energy E_{HB} and enthalpy ΔH_{HB} evaluated using Equations 2 and 1, respectively, are given in the last two columns.

H-bonded fragment[a]	R(H···O), Å	ρ_b (a.u.)	G_b (a.u.)	E_{HB} (kJ mol^{-1})	$-\Delta H_{HB}$ (kJ mol^{-1})
O(11)-H(11)…O(1)	2.095	0.0187	0.0151	**17.0**	**14.4**
O(11)-H(11)…O(6)	2.083	0.0187	0.0145	**16.3**	**16.03**
O(12)-H(12)…O(2)	1.815	0.0344	0.0255	**28.7**	**24.1**
O(21)-H(21)…O(11)	1.682	0.0479	0.0352	**39.6**	**30.7**
O(22)-H(22)…O(32)	1.684	0.0462	0.0350	**39.5**	**30.6**
O(31)-H(31)…O(21)[i]	1.692	0.0464	0.0350	**39.5**	**30.3**
O(32)-H(32)…O(42)	1.673	0.0487	0.0361	**40.7**	**31.3**
O(41)-H(41)…O(5)[ii]	1.801	0.0356	0.0262	**29.5**	**25.0**
O(42)-H(42)…O(4)[ii]	2.069	0.0198	0.0159	**17.9**	**16.64**
O(42)-H(42)…O(3)[ii]	1.907	0.0168	0.0131	**14.7**	**21.0**
Σ					

[a] See Figure S3.

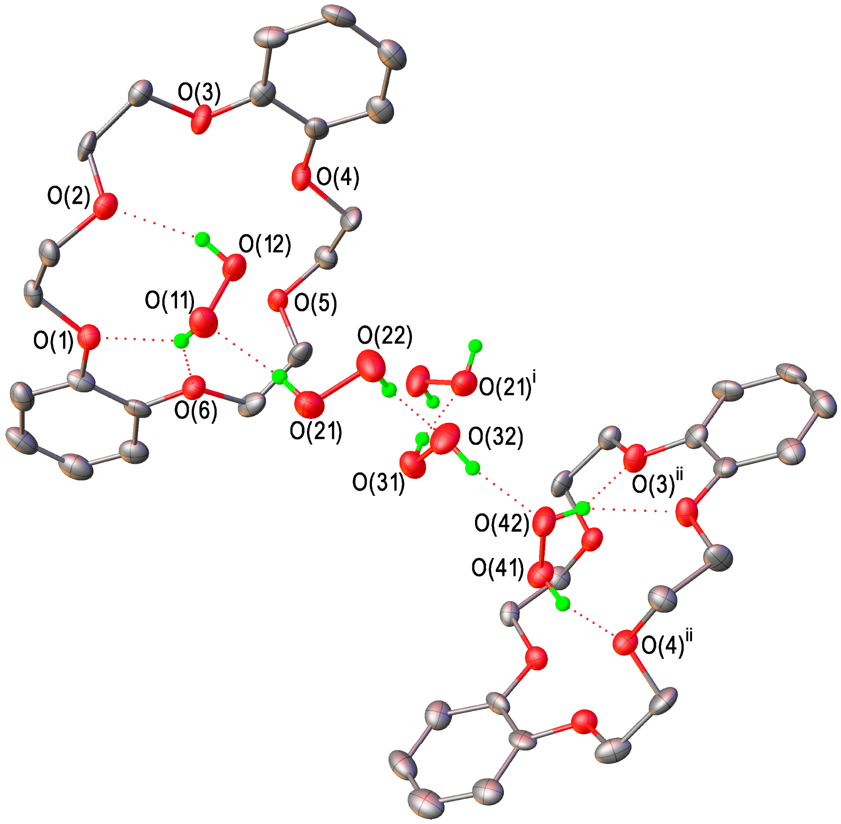

Figure S3. Part of the H-bond network in **III**. Displacement ellipsoids are shown at the 50% probability level. H atoms of macrocyclic ether are omitted for clarity. The H-bonds are given by the dashed lines. Symmetry operation: (i) -0.5+x,1.5-y,z; (ii) 0.5-x,0.5+y,-0.5+z.

Table S3b. The values of (H···O) distance, R(H···O), the electron density, ρ_b, and the local electronic kinetic energy density, G_b, at the O···O bond critical point obtained using partial B3LYP/6-31G** optimization of the **III** cluster. The H-bond energy E_{HB} and enthalpy ΔH_{HB} evaluated using Equations 2 and 1, respectively, are given in the last two columns.

H-bonded fragment[a]	R(H···O), Å	ρ_b (a.u.)	G_b (a.u.)	E_{HB} (kJ mol^{-1})	$-\Delta H_{HB}$ (kJ mol^{-1})
O(11)-H(11)…O(1)	2.215	0.0152	0.0131	**14.7**	**13.3**
O(11)-H(11)…O(6)	2.116	0.0172	0.0130	**14.6**	**15.3**
O(12)-H(12)…O(2)	1.904	0.0283	0.0215	**24.2**	**21.1**
O(21)-H(21)…O(11)	1.729	0.0435	0.0336	**37.8**	**28.3**
O(22)-H(22)…O(32)	1.733	0.0412	0.0325	**36.5**	**28.1**
O(31)-H(31)…O(21)[i]	1.839	0.0341	0.0264	**29.7**	**23.5**
O(32)-H(32)…O(42)	1.705	0.0457	0.0341	**38.3**	**29.5**
O(41)-H(41)…O(5)[ii]	1.893	0.0288	0.0217	**24.4**	**21.5**
O(42)-H(42)…O(4)[ii]	2.184	0.0161	0.0136	**15.3**	**13.9**
O(42)-H(42)…O(3)[ii]	2.122	0.0171	0.0128	**14.4**	**15.1**
Σ				**249.9**	**209.6**

Table **S4a**. B3LYP/6-31G** values of the (H···O) distance, *R*(H···O), the electron density, ρ_b, and the local electronic kinetic energy density, G_b, at the O···O bond critical point in creatine peroxosolvate **IV**. The H-bond energy E_{HB} and enthalpy ΔH_{HB} evaluated using Equations 2 and 1, respectively, are given in the last two columns.

H-bonded fragment[a]	*R*(H···O), Å	ρ_b (a.u.)	G_b (a.u.)	E_{HB} (kJ mol^{-1})	$-\Delta H_{HB}$ (kJ mol^{-1})
N(1)-H(12)…O(3)^{i}	1.900	0.0348	0.0259	**29.**	**21.2**
N(2)-H(21)…O(1)^{i}	1.863	0.0301	0.0235	**26.5**	**22.5**
N(2)-H(22)…O(2)^{iv}	1.923	0.0284	0.0202	**22.8**	**20.5**
O(1)-H(1)….O(6)^{ii}	1.692	0.0449	0.0331	**37.3**	**30.2**
O(2)-H(2)….O(6)	1.696	0.0451	0.0327	**36.8**	**30.0**
O(3)-H(3)…O(5)^{iii}	1.662	0.0497	0.0372	**41.9**	**31.9**
O(4)-H(4)…O(5)	1.787	0.0348	0.0259	**29.2**	**25.6**
Σ				**223.7**	**181.9**

[a)] the atomic numbering is given in Figure **S4**;

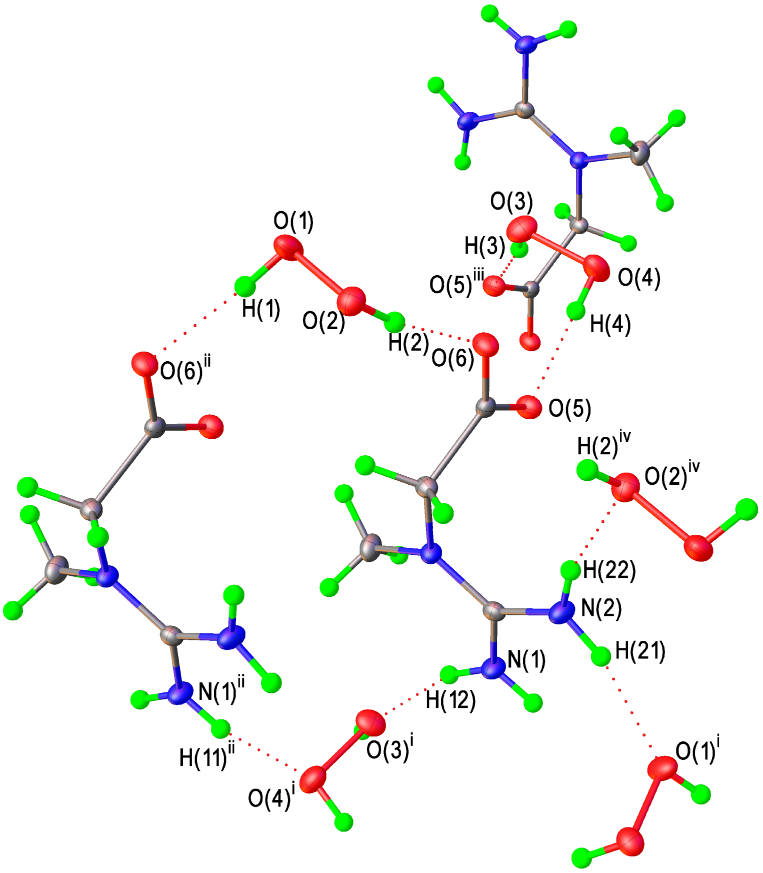

Figure S4. Part of the H-bond network in creatine peroxosolvate **IV**. Displacement ellipsoids are shown at the 50% probability level. H atoms of macrocyclic ether omitted for clarity. The H-bonds are given by the dashed lines. Symmetry operation: (i) x,1-y,-0.5+z; (ii) x,-1+y,z; (iii) 0.5-x,1.5-y, 1-z; (iv) 1-x,1-y,1-z.

Table S4b. The values of (H···O) distance, *R*(H···O), the electron density, ρ_b, and the local electronic kinetic energy density, G_b, at the O···O bond critical point obtained using partial B3LYP/6-31G** optimization of the **IV** cluster. The H-bond energy E_{HB} and enthalpy ΔH_{HB} evaluated using Equations 2 and 1, respectively, are given in the last two columns.

H-bonded fragment[a]	*R*(H···O), Å	ρ_b (a.u.)	G_b (a.u.)	E_{HB} (kJ mol^{-1})	$-\Delta H_{HB}$ (kJ mol^{-1})
N(1)-H(12)…O(3)[i]	1.972	0.0237	0.0194	**21.8**	**19.0**
N(2)-H(21)…O(1)[i]	1.868	0.0322	0.0245	**27.5**	**22.4**
N(2)-H(22)…O(2)[iv]	1.900	0.0274	0.0210	**23.6**	**21.2**
O(1)-H(1)….O(6)[ii]	1.708	0.0432	0.0333	**37.4**	**29.4**
O(2)-H(2)….O(6)	1.680	0.0465	0.0361	**40.6**	**30.9**
O(3)-H(3)…O(5)[iii]	1.747	0.0390	0.0296	**33.3**	**22.4**
O(4)-H(4)…O(5)	1.719	0.0431	0.0317	**35.6**	**28.8**
Σ				**219.9**	**174.1**

Table S5a. B3LYP/6-31G** values of the (H···O) distance, *R*(H···O), the electron density, ρ_b, and the local electronic kinetic energy density, G_b, at the O···O bond critical point in 3-phenylserine peroxosolvate **V** (refcode VILGAB). The H-bond energy E_{HB} and enthalpy ΔH_{HB} evaluated using Equations 2 and 1, respectively, are given in the last two columns.

H-bonded fragment[a]	*R*(H···O), Å	ρ_b (a.u.)	G_b (a.u.)	E_{HB} (kJ mol^{-1})	$-\Delta H_{HB}$ (kJ mol^{-1})
O(11)-H(11)…O(1)^{i}	1.553	0.06519	0.0488	**54.9**	**39.3**
O(12)^{ii}-H(12)^{ii}…O(11)	1.600	0.05922	0.0451	**50.7**	**35.6**
O(3)-H(13)…O(12)	1.955	0.02457	0.0182	**20.5**	**19.5**
N(1)^{i}-H(31)^{i}…O(2)	1.827	0.03281	0.0245	**27.5**	**23.9**
N(1)-H(32)…O(2)^{i}	1.897	0.02878	0.0218	**24.5**	**21.3**
N(1)^{iii}-H(33)^{iii}…O(1)	1.837	0.03470	0.02411	**27.2**	**23.5**
Σ	-	-	-	**205.5**	**163.1**

[a] See Figure **S5**.

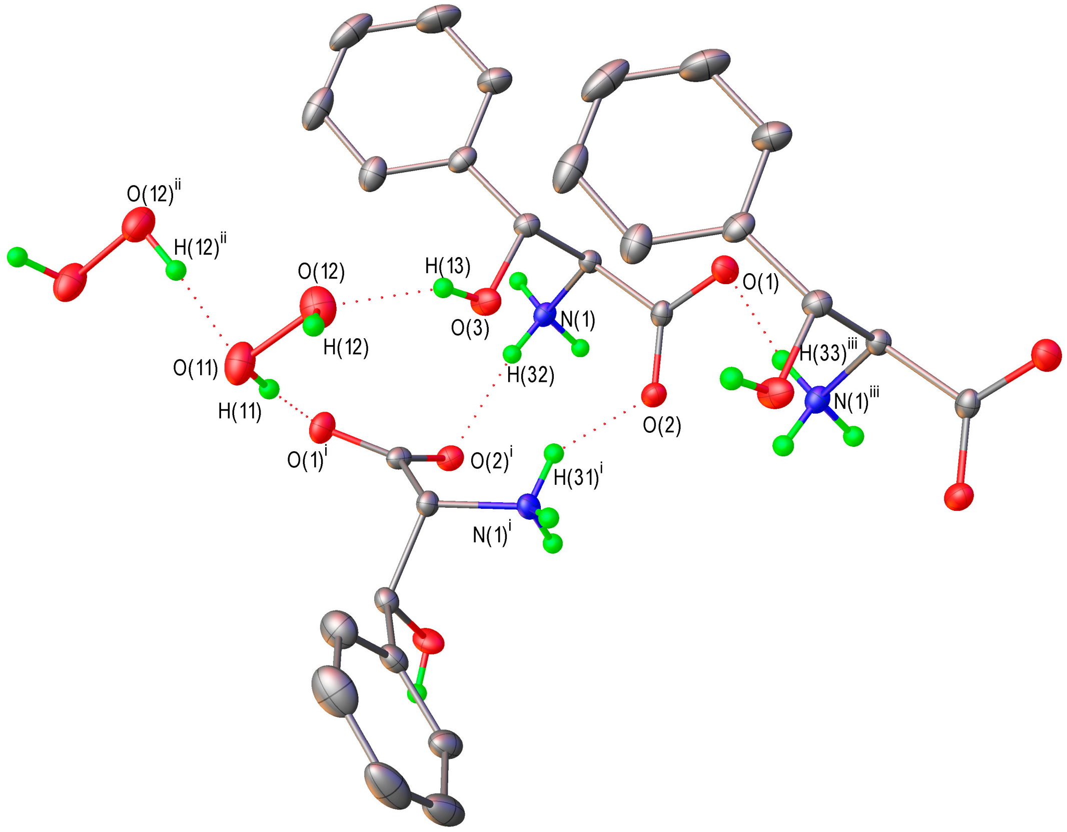

Figure S5. Part of the H-bond network in 3-phenylserineperoxosolvate **V**. Displacement ellipsoids are shown at the 50% probability level. Only «active» hydrogen atoms are shown. The H-bonds are given by the dashed lines. Symmetry operation: (i) -x,y,-z; (ii) 0.5-x,0.5-y,0.5+z; (iii) x,y,-1+z.

Table S5b. The values of (H···O) distance, R(H···O), the electron density, ρ_b, and the local electronic kinetic energy density, G_b, at the O···O bond critical point obtained using partial B3LYP/6-31G** optimization of the **V** cluster. The H-bond energy E_{HB} and enthalpy ΔH_{HB} evaluated using Equations 2 and 1, respectively, are given in the last two columns.

H-bonded fragment[a]	R(H···O), Å	ρ_b (a.u.)	G_b (a.u.)	E_{HB} (kJ mol^{-1})	$-\Delta H_{HB}$ (kJ mol^{-1})
O(11)-H(11)…O(1)^{i}	1.589	0.05871	0.0464	**52.2**	**36.6**
O(12)^{ii}-H(12)^{ii}…O(11)	1.688	0.04724	0.0379	**42.6**	**30.5**
O(3)-H(13)…O(12)	1.967	0.02462	0.0175	**19.7**	**19.1**
N(1)^{i}-H(31)^{i}…O(2)	1.826	0.03497	0.0228	**25.6**	**24.0**
N(1)-H(32)…O(2)^{i}	1.798	0.03504	0.0251	**28.2**	**25.1**
N(1)^{iii}-H(33)^{iii}…O(1)	1.869	0.03289	0.0222	**25.0**	**22.3**
Σ	-	-	-	**193.3**	**157.6**

Table S6. Distances between the atoms involved in the formation of intermolecular H-bonds *R*(O···O) and *R*(H···O) in **I**[a)], obtained using periodic DFT computations (PBE-D3/6-31G** and B3LYP/6-31G**). Theoretical values of the enthalpy, ΔH_{HB} of intermolecular H-bonds evaluated using Equation (1) are given in the last two columns.

Fragment [b)]	*R*(O···O)/*R*(H···O), Å			ΔH_{HB}, kJ mol^{-1}	
	X-ray	B3LYP	PBE-D3	B3LYP	PBE-D3
O(4A)-H(4A)…O(1)	2.8404 (2.043)	2.835 (1.896)	2.828 (1.883)	21.4	21.8
O(5A)-H(5A)…O(3)	2.8032 (1.995)	2.775 (1.807)	2.768 (1.788)	24.7	25.5
O(6)-H(6)…O(2)	2.7613 (1.911)	2.749 (1.783)	2.746 (1.766)	25.8	26.5
O(7)-H(7)…O(6)^{iii}	2.8469 (2.114)	2.801 (1.930)	2.776 (1.874)	20.2	22.1
O(7)-H(7)…O(5A)^{iv}	3.0398 (2.527)	3.098 (2.502)	- (-)	9.2	-
O(8)-H(8)…O(7)	2.8498 (2.05)	2.830 (1.854)	2.827 (1.840)	22.9	23.4

a) to get rid of imaginary frequencies, the space symmetry group of the crystal **I** was reduced to P-1;

b) the atomic numbering is given in **Figure S1**;

Table S7. Distances between the atoms involved in the formation of intermolecular H-bonds *R*(O···O) and *R*(H···O) in **II**, obtained using periodic DFT computations (PBE-D3/6-31G** and B3LYP/6-31G**). Theoretical values of the enthalpy, ΔH_{HB} of intermolecular H-bonds evaluated using Equation (1) are given in the last two columns.

Fragment [a)]	*R*(O···O)/*R*(H···O), Å			ΔH_{HB}, kJ mol^{-1}	
	X-ray	B3LYP	PBE-D3	B3LYP	PBE-D3
O(1)^{i}-H(1)^{i}…O(11)	2.667 (1.81)	2.665 (1.677)	2.605 (1.659)	31.1	32.1
O(2)-H(2)…O(11)	2.698 (1.80)	2.693 (1.700)	2.686 (1.678)	29.8	31.0
O(3)^{ii}-H(3)^{ii}…O(12)	2.761 (1.89)	2.719 (1.725)	2.703 (1.694)	28.5	30.1
O(4)-H(4)…O(12)	2.707 (1.81)	2.668 (1.706)	(1.689)	29.5	30.4
O(5)-H(5)…O(11)	2.667 (1.77)	2.659 (1.663)	(1.645)	31.9	32.9
O(6)^{iii}-H(6)^{iii}…O(12)	2.773 (1.87)	2.758 (1.767)	2.742 (1.737)	26.5	27.9

a) the atomic numbering is given in **Figure S2**;

Table S8. Distances between the atoms involved in the formation of intermolecular H-bonds R(X···O) and R(H···O) in creatine diperoxosolvate **IV**, where X = N or O, obtained using periodic DFT computations (PBE-D3/6-31G** and B3LYP/6-31G**). Theoretical values of the enthalpy, ΔH_{HB} of intermolecular H-bonds evaluated using Equation (1) are given in the last two columns.

Fragment [a)]	R(X···O)/R(H···O), Å			ΔH_{HB}, kJ mol^{-1}	
	X-ray	B3LYP	PBE-D3	B3LYP	PBE-D3
N(1)-H(11)…O(4)^{v} [b)]	2.991 (2.328)	-	-		
N(1)-H(12)…O(3)^{i}	2.860 (2.011)	2.900 (1.900)	2.896 (1.883)	21.2	21.8
N(2)-H(21)…O(1)^{i}	2.854 (1.977)	2.852 (1.863)	2.844 (1.847)	22.5	23.1
N(2)-H(22)…O(2)^{iv}	2.879 (2.026)	2.923 (1.923)	2.907 (1.894)	20.5	21.4
O(1)-H(1)….O(6)^{ii}	2.689 (1.841)	2.689 (1.692)	2.681 (1.669)	30.2	31.5
O(2)-H(2)….O(6)	2.665 (1.790)	2.685 (1.696)	2.681 (1.675)	30.0	31.2
O(3)-H(3)…O(5)^{iii}	2.718 (1.866)	2.653 (1.662)	2.652 (1.647)	31.9	32.8
O(4)-H(4)…O(5)	2.698 (1.829)	2.769 (1.787)	2.772 (1.777)	25.6	26.0

[a)] the atomic numbering is given in Figure S4;

[b)] strongly nonlinear fragment N-H···O, angle N(1)-H(11) ···O(4)^{v} less than 140 deg. Symmetry operation (v) x, 2-y, -0.5+z.

Table S9. X-ray structure determination summary

Compound	**I**	**II**	**III**	**IV**
Empirical formula	$C_{12}H_{24}O_6$ •5(H_2O_2)	$C_{18}H_{36}N_2O_8$ •3(H_2O_2)	$C_{20}H_{24}O_6$ •4(H_2O_2)	$C_4H_9N_3O_2$ •2(H_2O_2)
F_w	434.39	510.54	496.46	199.17
Crystal system	Monoclinic	Triclinic	Orthorhombic	Monoclinic
Space group	*P*2/*c*	*P*-1	$Pna2_1$	*C2/c*
a/Å	8.2579(7)	10.0806(7)	11.402(2)	21.7326(4)
b/Å	7.2124(6)	11.4888(6)	11.308(2)	4.9666(1)
c/Å	17.8401(15)	12.3905(7)	17.896(3)	16.3358(3)
$\alpha/^o$	90	106.013(2)	90	90
$\beta/^o$	98.496(1)	109.717(2)	90	91.6775(8)
$\gamma/^o$	90	97.943(2)	90	90
V/Å^3	1050.9(2)	1255.32(13)	2307.3(7)	1762.48(6)
Z	2	2	4	8
F(000)	468	552	1056	848
d_{calc}/g•cm^{-3}	1.373	1.351	1.429	1.501
μ/mm^{-1}	0.129	0.116	0.122	0.140
T/K	150	150	120	120
Data collected	11894	6035	11980	10461
Unique data (R_{int})	3066 (0.019)	6035 (0.040)	2114 (0.137)	2532 (0.025)
Reflections with $I>2\sigma(I)$	2613	4515	1261	2327
θ range/°	2.31-30.00	1.86-28.00	2.28-25.04	2.50-30.00
No of variables	214	331	331	170
R_1 [$I>2\sigma(I)$]	0.033	0.048	0.045	0.034
wR_2 (all data)	0.094	0.114	0.113	0.085
GOF	1.040	1.049	1.005	1.033
$\Delta\rho_{max,min}$/e Å^{-3}	0.34 / -0.13	0.30 / -0.21	0.28 / -0.25	0.37 / -0.18
